# Stretching Method-Based Operational Modal Analysis of An Old Masonry Lighthouse

**DOI:** 10.3390/s19163599

**Published:** 2019-08-19

**Authors:** Emmanouil Daskalakis, Christos G. Panagiotopoulos, Chrysoula Tsogka, Nikolaos S. Melis, Ioannis Kalogeras

**Affiliations:** 1 Vancouver Community College, 1155 E Broadway, Vancouver, BC V5T 4V5, Canada; 2 Institute of Applied and Computational Mathematics, Foundation for Research and Technology, Hellas, N. Plastira 100, Vassilika Vouton, GR-700 13 Heraklion, Crete, Greece; 3 Department of Applied Mathematics, University of California, Merced, 5200 North Lake Road, Merced, CA 95343, USA; 4 Institute of Geodynamics, National Observatory of Athens, P.O. Box 20048, GR-118 10 Athens, Greece

**Keywords:** SHM, stretching method, model updating

## Abstract

We present in this paper a structural health monitoring study of the Egyptian lighthouse of Rethymnon in Crete, Greece. Using structural vibration data collected on a limited number of sensors during a 3-month period, we illustrate the potential of the stretching method for monitoring variations in the natural frequencies of the structure. The stretching method compares two signals, the current that refers to the actual state of the structure, with the reference one that characterizes the structure at a reference healthy condition. For the structure under study, an 8-day time interval is used for the reference quantity while the current quantity is computed using a time window of 24 h. Our results indicate that frequency shifts of 1% can be detected with high accuracy allowing for early damage assessment. We also provide a simple numerical model that is calibrated to match the natural frequencies estimated using the stretching method. The model is used to produce possible damage scenarios that correspond to 1% shift in the first natural frequencies. Although simple in nature, this model seems to deliver a realistic response of the structure. This is shown by comparing the response at the top of the structure to the actual measurement during a small earthquake. This is a preliminary study indicating the potential of the stretching method for structural health monitoring of historical monuments. The results are very promising. Further analysis is necessary requiring the deployment of the instrumentation (possibly with additional instruments) for a longer period of time.

## 1. Introduction

This work takes place in the general framework of structural health monitoring (SHM) of historic monuments [1,2,3,4]. Ideally, the monitoring system should provide a quick assessment of the state of the structure without requiring a long training period while using only a limited number of instruments and computational resources. Several vibration-based output only modal identification techniques are available in the literature for this purpose (see for example [5,6,7] and references therein). Since the estimated modal frequencies depend highly on changing environmental conditions (such as the temperature, and the wind direction/strength), the use of statistical methods is necessary so as to remove this dependance and provide reliable indicators of structural damage [8,9,10,11,12,13,14,15]. We also refer to [16] for a review on data normalization procedures for addressing the issue of separating structural changes of interest from operational and environmental variations. These statistical techniques, however, often require heavy computations and a long training period, of the order of one year, before the monitoring system can be operational [11]. To address this problem, we have introduced in [17] the Stretching Method (SM) which, in the context of SHM, allows the estimation of small shifts in the natural frequencies of a structure in a simple and efficient way without requiring a long training period.

The stretching method has been successfully used to detect and monitor changes in the substratum of the earth [18]. To do so, SM compares two waveforms, the current and the reference one, both obtained by cross-correlating ambient noise recordings. These two waveforms characterize the current and reference state of the substratum (e.g., the velocity with which waves propagate in the Earth) [19,20]. The difference between the two waveforms is the time-window over which the cross-correlations are averaged. A large time window is required to build the reference waveform, while a smaller one is used to obtain the current waveform. SM determines the amount of stretching that the current waveform needs so as to maximize its correlation with the reference waveform. This stretching corresponds to a relative change in the velocity with which waves propagate in the substratum.

The application of the stretching method in the structural health monitoring context was considered in [17]. There, SM was adequately modified so as to detect and monitor changes in the natural frequencies of a structure. In contrast to seismology, the cross-correlations instead of being compared in the time domain, they are processed in the frequency domain using the Fourier transform. In the SHM context, we can interpret the time-window over which the reference cross-correlation is computed as the training period of the method which is typically 7–9 days while the time needed to compute the current waveform determines the damage warning time shown to be 12–24 h [17].

In this paper we apply this SM-based methodology on output only vibration data collected on the Egyptian lighthouse located at the port of Rethymnon on the island of Crete in Greece. SM is successfully used to identify the natural frequencies of the structure related to flexural bending modes. However, due to lack of sufficient data, other modes such as axial or torsional, while shown to be present in the signals, were not vigorously identified. Based on a 2-hour time averaging period for the current quantity, we carry out an analysis of the fluctuations for the first two natural frequencies of the structure. Our analysis indicates strong dependence on temperature and also on wind direction. The fluctuations in the natural frequencies are reduced by increasing the time-averaging window over which the current waveform is computed. A time window of 24-hours seems to provide sufficiently accurate estimates of artificially imposed frequency shifts. This is determined with a sensitivity analysis that allows us to estimate with what accuracy different levels of frequency shifts in the natural frequencies can be detected. This analysis shows in particular that our methodology can be safely used for recovering frequency shifts as small as 1% when using 1-day intervals for averaging cross-correlations to compute the current quantity.

We also propose a simple model that is calibrated to match the estimated natural frequencies using model updating techniques. Generally speaking, a mathematical or numerical model may be used to simulate the behaviour and response under certain excitation of a structure. This model is often built using the finite element method. In practice, however, several properties of the real system are unknown or uncertain (i.e., material and geometric properties, boundary conditions, excitation), for which, inevitably, conjectures have to be made. Moreover, due to lack of knowledge or other restrictions, often simplifying modeling assumptions regarding the structure are required or implicitly made [21]. Due to these uncertainties, it is often better to define a simple initial model amenable to easy and efficient modifications. Furthermore, a simple surrogate model with a limited number of degrees of freedom is more appropriate to be used for possible near real time results as an output of some simulation that could be executed to embedded systems (e.g., microprocessor).

Here, we use a simple model that consists of two independent cantilever beam systems. The first refers to the east–west and the second to the south–north directions. Using model updating techniques [22] we seek to estimate elasticity (Young’ modulus) and inertia (mass density) distribution over the length of the beams so as to match the model’s eigenfrequencies to the estimated natural frequencies of the structure. The calibrated model, although very simple, matches (relatively well) the response of the structure to a small earthquake. This calibrated model is also used to provide possible damage scenarios associated with specific measured shifts.

The remainder of the paper is structured as follows. In Section 2, we present a brief review of the stretching method and explain how it can be used for operational modal analysis. Then, the application of the method and the analysis of the data collected on the Rethymnon lighthouse is carried out in Section 3. Section 4 is concerned with the model updating technique and Section 5 contains our conclusions.

## 2. Methodology Review: The Stretching Method

The Stretching Method (SM) is widely used in passive imaging applications mostly related to Geophysics [23,24,25]. The key idea of the method is to estimate how much we need to stretch a signal, referred to as current, so as to maximize its correlation coefficient with another signal, the reference. In Geophysics, the signals on which SM is applied are time domain cross correlations of seismic noise recordings. The reference signal characterizes the medium with no variations while the current characterizes the medium in its present condition. The difference between the two signals is the time interval over which the cross-correlations are averaged. Longer time intervals are required to obtain the reference quantity while for the current signal, the cross-correlations are averaged over a small time window. The resulting stretching parameter corresponds to the relative velocity change dv/v of the medium. Recently, SM was successfully used in the context of Structural Health Monitoring (SHM). The methodology is briefly explained below, for more details we refer the interested reader to [17].

Let $u_{k}\left(t,x_{l}\right)$ and $u_{m}\left(t,x_{n}\right)$ be the recordings of the acceleration due to random excitations in the *k*-th and *m*-th directions at locations $x_{l}$ and $x_{n}$, respectively. Combination of direction and location defines a specific degree of freedom, $u^{i}\left(t\right)=u_{k}\left(t,x_{l}\right)$ and $u^{j}\left(t\right)=u_{m}\left(t,x_{n}\right)$. We use these recordings to compute the empirical cross-correlation function over the time interval [0,T],

(1) $$C_{T}^{i,j}\left(\tau \right)=\frac{1}{T}\int_{0}^{T}u^{i}\left(t\right)u^{j}\left(t+\tau \right)dt,$$

with τ denoting the lag-time. The cross-correlation function $C_{T}$ is a self averaging quantity, which means that it is independent of the random excitation, provided that the averaging time window is large enough. More precisely, it can be shown that $C_{T}$ converges to the average quantity $C^{\mathrm{av}}$,

(2) $$C_{T}^{i,j}\left(\tau \right)\overset{T\to \infty }{⟶}C_{\mathrm{av}}^{i,j}\left(\tau \right),$$

where the average is defined with respect to the realizations of the random excitations

(3) $$C_{\mathrm{av}}^{i,j}\left(\tau \right)=\langle C_{T}^{i,j}\left(\tau \right)\rangle .$$

In the SHM context, SM is not applied directly to the time domain cross-correlations but rather to their Fourier transforms. Assuming that the excitations behave like random white noise and the structure is a linear and time invariant system, the elements of the cross-correlation matrix in the frequency domain correspond to power spectral densities. In this case, the resulting stretching parameter corresponds to the relative change dν/ν in the natural frequencies of the structure. More precisely, we form the matrix

$${\left[A^{\#}\left(\nu \right)\right]}_{i,j}=A_{i,j}^{\#}\left(\nu \right),\;\;\#=r,\;c$$

where $A_{i,j}^{\#}\left(\nu \right)$ is the Fourier transform of the reference (#=r) or current (#=c) empirical cross-correlation (1) computed for sensors *i* and *j*, 1≤i,j≤n, where *n* denotes the number of the available (measured) degrees of freedom,

(4) $$A_{i,j}^{\#}\left(\nu \right)=\int C_{T}^{i,j}\left(\tau \right)e^{ı2\pi \tau \nu }d\tau .$$

The matrix $A^{\#}\left(\nu \right)$ is symmetric so it admits a symmetric singular value decomposition (SVD) of the form,

(5) $$A^{\#}\left(\nu \right)=U^{\#}\left(\nu \right)\Sigma^{\#}\left(\nu \right)U^{{}^{\#},T}\left(\nu \right).$$

The matrix $\Sigma^{\#}\left(\nu \right)$ is a real diagonal matrix with the singular values $\sigma_{1}^{\#}\left(\nu \right),\ldots \sigma_{n}^{\#}\left(\nu \right)$ placed on the diagonal while the columns $u_{1}^{\#}\left(\nu \right),\ldots ,u_{n}^{\#}\left(\nu \right)$ of the matrix $U^{\#}\left(\nu \right)$ are the corresponding singular vectors. Here $U^{{}^{\#},T}$ denotes the transpose of the matrix $U^{{}^{\#}}$. The frequencies at which the first singular value admits peaks correspond to the natural frequencies of the structure while the first singular vector at the corresponding frequency is identified as the modal shape associated to this natural frequency (see Section 10.3.3 of [26]).

To monitor variations in the natural frequencies with the SM method we maximize the correlation coefficient

(6) $$C_{s}\left(\Delta \nu \right)=\frac{\int_{\nu_{1}}^{\nu_{2}}\sigma_{1}^{c}\left(\nu +\Delta \nu \right)\sigma_{1}^{r}\left(\nu \right)d\nu }{\sqrt{\int_{\nu_{1}}^{\nu_{2}}{\left(\sigma_{1}^{c}\left(\nu +\Delta \nu \right)\right)}^{2}d\nu \int_{\nu_{1}}^{\nu_{2}}{\left(\sigma_{1}^{r}\left(\nu \right)\right)}^{2}d\nu }},$$

where $\sigma_{1}^{r}$ and $\sigma_{1}^{c}$ are the reference and current largest singular values of the matrices $A^{r}$ and $A^{c}$ respectively. The interval over which the optimization is performed is centered around each of the detected natural frequencies of the structure. The discretization is adaptively refined during the optimization process so as to provide an efficient solution. The result of this procedure provides a value for Δν for each current quantity. Tracking the changes Δν over time could help us detect potential structural damage [26].

## 3. Application to the Rethymnon Egyptian Lighthouse

In this section we apply the stretching method to structural vibration data of the Rethymnon lighthouse.

### 3.1. Structural Elements of the Lighthouse

**Figure 1:**
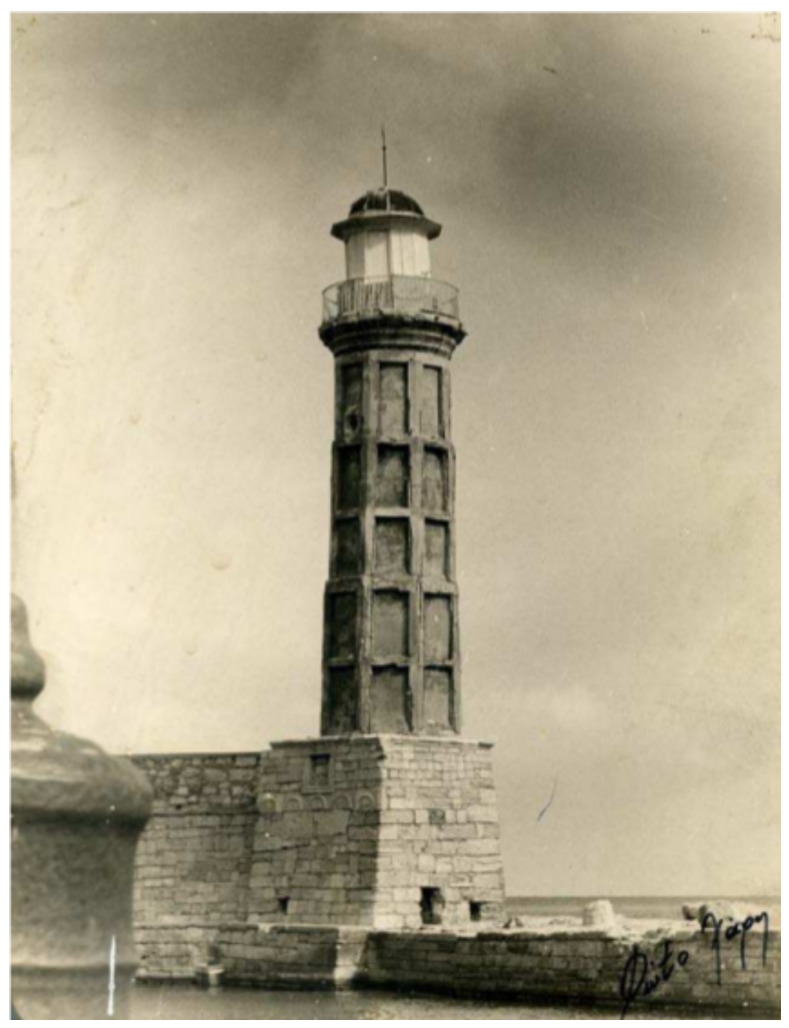
An older shot of the Egyptian lighthouse of Rethymno.

The Egyptian lighthouse of Rethymnon is a masonry building constructed in 1838 while still Crete (Greece) was an Ottoman province under Egyptian control. The lighthouse came under the supervision of the French Lighthouse Company in 1864. It was operating until 1962. In 1930, for strengthening purposes, concrete reinforcement was used, as it may also be seen in the photo of Figure 1.

**Figure 2:**
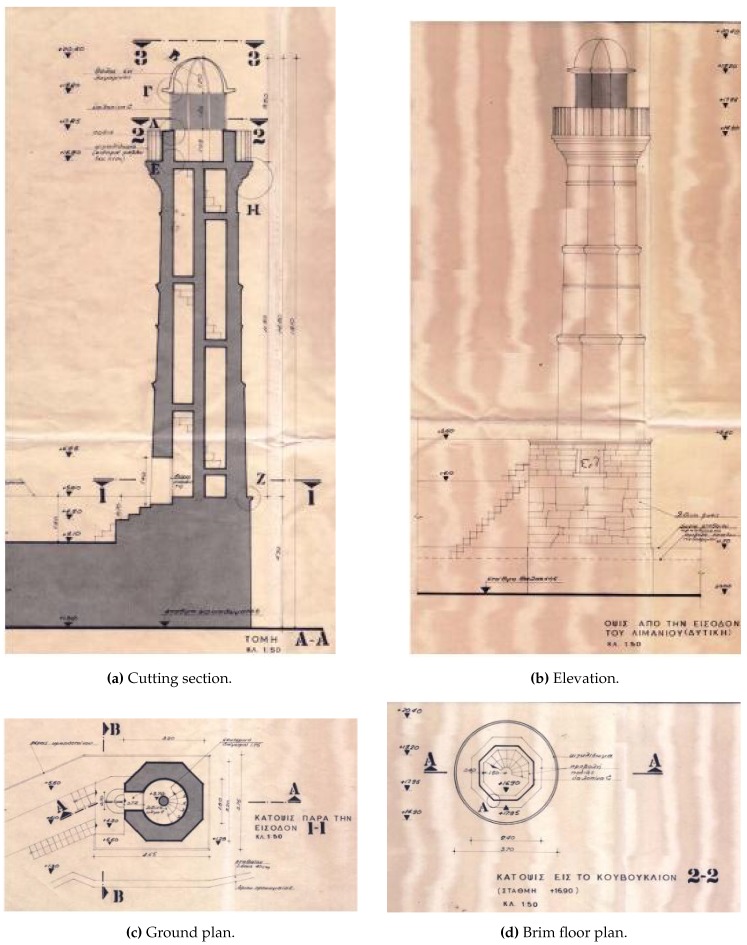
Sketches of an older rehabilitation study.

Elements of the geometric properties of the lighthouse have been adopted from data of an older rehabilitation study (1973, Lampakis, Civil Engineer), shown in Figure 2.

Based on this study, and using a preliminary simplified model, we initially assume a length *L* = 15.6 m and consider the lighthouse as a hollow cylinder of constant cross section having internal radius $r_{i}$ = 1.2 m and external $r_{e}$ = 1.8 m. The material is assumed homogeneous with a modulus of Elasticity *E* = 5 GPa and mass density ρ = 2.5 tn/m${}^{3}$. These values belong in a range typical for masonry of historical buildings and give a good agreement between the model predicted eigenfrequencies (Section 3.3.1) and those estimated from the measurements (see Section 3.3.2). In Section 4.1.1, we use a heterogeneous cantilever beam model and estimate the distribution of its elasticity modulus and mass density by minimizing the misfit between the model predicted and the measured eigenfrequencies.

### 3.2. Instrument Placement

The deployment of the accelerographic instruments started in May 2017, after the Rethymno Ephorate of Antiquities expressed interest within the frame of the EU H-2020 STORM project. NOAIG installed two digital three-component accelerographs of CMG-5TDE type (Guralp Systems Ltd, 24 bit at 200 s/s, continuous local recording, time-stamped via GPS) at the top and the basement of the lighthouse. Care was taken for the installations to have the same orientation and to be at the same vertical axis of the lighthouse, in order to minimize possible effects from the directivity of the ground motion energy propagation and from the rotation of the body of the lighthouse.

The two instruments were recording acceleration in three directions (North, East and vertical) for a period of three months in separate 2-hour long files. We have a total of n=6 degrees of freedom recorded corresponding to three directions at the top and three directions at the bottom of the lighthouse. Although the two instruments were vertically aligned, since the diameter of the lighthouse is changing with height, the alignment is not perfect.

In addition, we included in our study meteorological data (temperature, wind speed and wind direction), which were kindly offered by the Institute for Environmental Research and Sustainable Development of the National Observatory of Athens, that has developed and operates a network of automated weather stations to monitor weather conditions in Greece. Data were acquired from the weather station permanently deployed at the Municipal Enterprise of Water and Sewerage Supplies of Rethymno. The station is situated close to the coast and the Lighthouse under investigation. The meteorological station includes a Davis Instruments, type: Vantage Pro2 Wired Fan-Aspirated, weather monitoring instrument. Further detailed information on the station configuration and the produced data, as well as the NOA meteo network can be viewed in [27,28].

### 3.3. Modal Recognition

Using the limited number of sensors, that is two tri-axial (3D) sensors, it is possible to estimate the natural frequencies (eigenfrequencies) of the structure while for the corresponding eigenfunctions we only get a very crude estimate (i.e., their values at the two measurement locations).

#### 3.3.1. Modal Recognition—Theoretical Expectations

Approximating the lighthouse tower as a cantilever beam of constant cross section and adopting the Euler–Bernoulli assumptions, we may analytically express the respective eigensystem and obtain an estimation for the eigenfrequencies and eigenfunctions pairs. We believe that according to the instruments’ placement as described in Section 3.2, we could, with certainty, measure only flexural due to bending modes. Other modes such as axial or torsional may also exist, however, due to lack of sufficient data, we cannot safely confirm that.

The moment of inertia is taken to be $I=\pi (r_{e}^{4}-r_{i}^{4})/4$ = 6.616 m${}^{4}$. The eigenfunctions corresponding to flexural vibration of the cantilever beam are (see for example in [29]),

(7) $$\begin{array}{c} \phi_{n}\left(x\right)=\cosh\lambda_{n}x-\cos\left(\lambda_{n}x\right)+\frac{\sinh\left(\lambda_{n}L\right)-\sin\left(\lambda_{n}L\right)}{\cosh\left(\lambda_{n}L\right)-\cos\left(\lambda_{n}L\right)}\left(\sinh\left(\lambda_{n}x\right)-\sin\left(\lambda_{n}x\right)\right) \end{array}$$

while, eigenfrequencies $\omega_{n}$ are given as,

(8) $$\begin{array}{c} \omega_{n}=\lambda_{n}^{2}\sqrt{\frac{EI}{mL^{4}}} \end{array}$$

where, the first two coefficients $\lambda_{n}L$ are given as 1.8751 and 4.6941, respectively. Furthermore, m=ρA = 14.137 tn/m, the beam’s mass per length, and $A=\pi (r_{e}^{2}-r_{i}^{2})$ = 5.655 m${}^{2}$, the cross section area. The coefficient of flexural rigidity is EI = 33080970.64 kNm${}^{2}$. The first two flexural (angular) eigenfrequencies $\omega_{n}$, corresponding eigenperiods $T_{n}=2\pi /\omega_{n}$, and ordinary eigenfrequencies $\nu_{n}=1/T_{n}$, are calculated as:

$$\begin{array}{ccc} \omega_{1}=22.101\;\mathrm{rad}/\sec,\; & T_{1}=0.284\;\sec,\; & \nu_{1}=3.517\;\mathrm{Hz},\\ \omega_{2}=138.504\;\mathrm{rad}/\sec,\; & T_{2}=0.045\;\sec,\; & \nu_{2}=22.043\;\mathrm{Hz}.\; \end{array}$$

#### 3.3.2. Modal Recognition—Signal Processing

To apply the SM method, we first compute the empirical cross-correlations according to Equation (1) for all different pairs of measurements. The reference quantity is computed by averaging the recordings over the first 8 days of acquisition. The current quantity will be computed either using a 2-hour interval or a 24-hour interval. The idea is to use for the current quantity the smallest possible window that guarantees detection of small shifts in Δν with high accuracy. This allows for assessing any structural damage of the structure as soon as possible. As illustrated in Figure 4, Δν measurement has a large variance due to temperature variations when a time-window of 2-hours for the current quantity is used. To chose the adequate size of the time window for the current quantity, a sensitivity analysis is carried out (results shown in Figures 7 and 8). This consists of artificially inserting a frequency shift in the monitoring data and then trying to recover it using SM. Our analysis suggests that the 24-hour interval is a good time window for detecting a frequency shift of 1% with high accuracy.

Then, the Fourier transform of the current and reference cross-correlations is computed and the corresponding 6×6 matrices are formed. Since the values of these matrices depend on the frequency, their singular value decomposition, performed frequency by frequency, provides singular values that are frequency dependent as well. Our analysis uses the first singular value of the corresponding matrix as reference and current quantity in (6).

**Figure 3:**
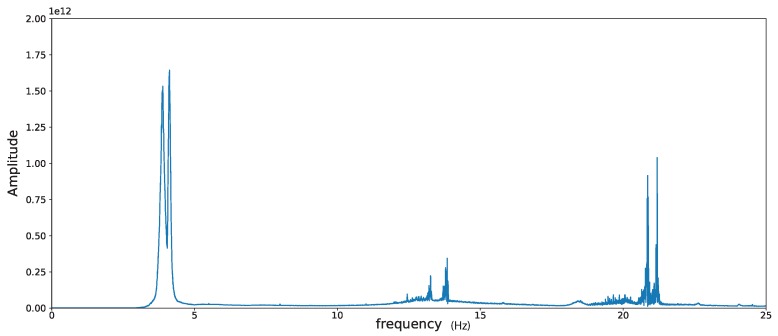
The first singular value of the reference cross-correlation matrix (computed using the first 8 days of acquisition) as a function of frequency (in Hz), $\sigma_{1}^{r}\left(\nu \right)$. The estimated values for the first seven natural frequencies of the structure are given in Table 1.

To estimate the modes of the structure under examination, we look at the first singular value of the reference cross-correlation matrix as a function of frequency. The frequencies at which this function admits peaks are identified as structural modes. By looking at the plot in Figure 3 (i.e., the first singular value of the reference cross-correlation matrix as a function of frequency), we have identified the eigenfrequencies given in Table 1. We do not have an interpretation for the modes 3–5. We believe that they correspond to torsional and axial modes, however, not appropriately measured due to the instruments’ placement.

#### 3.3.3. Modal Recognition—Theoretical Modal Values vs Recovered Modes and Conclusions

By comparing the results of Section 3.3.1 and Section 3.3.2, we observe that quite good agreement has been obtained between theoretically expected, under certain rough approximations, and measured values for flexural modes of the structure. There are three estimated eigenfrequencies in the neighbourhood of 13 Hz, which due to lack of sufficient data, could not be safely recognized and attributed with specific physical meaning, while they seem to be related with torsional and axial response or other secondary motion of non-structural components on which instruments are located.

### 3.4. Δν Measurements

**Figure 4:**
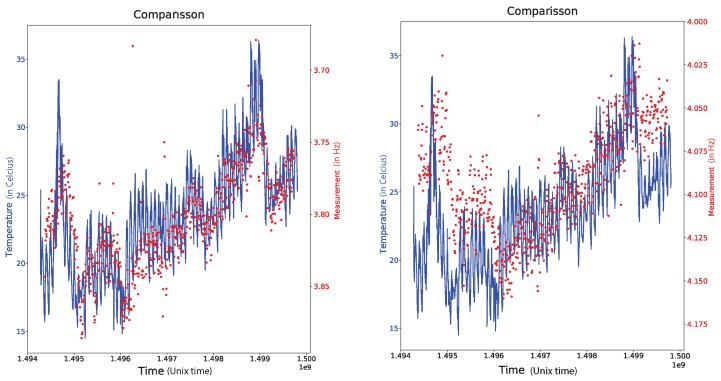
Comparison between the Δν measurement and the temperature for the first (**left**) and second (**right**) mode.

We focus our attention now on the first two identified modes and look at their variations as a function of time over the acquisition period. It is well known that the variations of Δν over time are mainly driven by variations in temperature [30]. To examine if the same holds here, we compare in Figure 4 the variations Δν for the first two modes with the temperature variations over the same time period.

With a first look at the results (see Figure 4), we observe that the measurement that corresponds to the first mode seems to be highly correlated with the temperature while the measurement of the second mode is less correlated with the temperature. More precisely, the measurement seems to be differently correlated at the very beginning and the very end of the measurement period in comparison with the period in between.

The explanation of this behaviour relies on the source of the excitation for these two modes. The two modes correspond to different flexural vibrations and, thus, each mode excitation is expected to depend on the wind direction.

**Figure 5:**
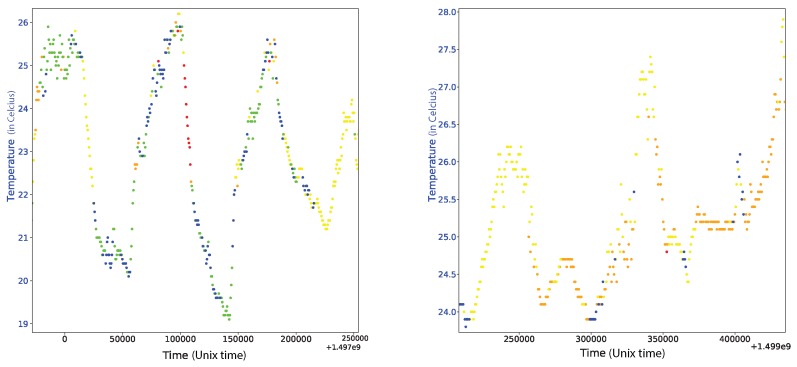
The temperatures for two different days and the correlation with the wind direction. The direction of the wind is represented by the different dot colors with blue to be a North–South direction and with red a West–East direction. On the left we have a couple of days at the middle of the measurement period and on the right a couple of days at the end of the measurement period.

In Figure 5, we plot the direction of the wind for two different time segments in the three month acquisition period. On the left we have a few days with wind that varies widely in direction and thus guarantees an excitation source for both modes measured in Figure 4. On the right we have a time period with winds that barely vary in direction (mostly East–West winds) and thus do not provide sufficient excitation for the second mode. Although the natural frequencies of the structure do not depend on the wind direction our ability to measure them using vibration data does depend on it. Indeed the SM assumes measurements due to white isotropic noise. If the noise is not isotropic, it affects the ability of SM to recover the measurement for Δν. This explains why the second mode does not correlate with the temperature in the same way as the first mode does during the periods that the wind is mostly along a single direction.

**Figure 6:**
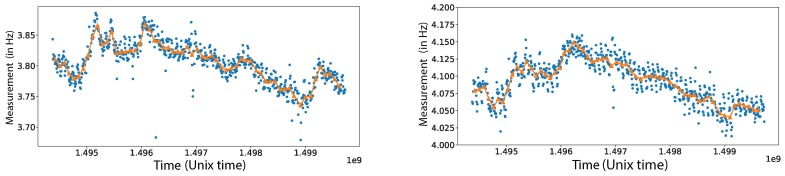
The estimated values for the first (right) and the second (left) eigenmode using for the current quantity a time window of 2 h (blue dots) and 24 h (orange triangles). The reference quantity is the same for both plots and is computed using the first 8 days of data.

To decrease the variations of Δν so as to be able to asses damage associated with frequency shifts in the structural modes we propose to compute the current quantity using a time-window of 24-hours. This significantly reduces the variations of Δν as can be seem in Figure 6.

To illustrate that SM can effectively allow us to detect small permanent shifts in the structural modes that could be associated with a damage, we artificially insert a frequency shift in the monitoring data and then try to recover it. Over the assumed period over which there is damage, we define the modified singular values ${\tilde{\sigma }}_{i}\left(\nu \right)$ as follows,

(9) $${\tilde{\sigma }}_{i}\left(\nu \right)=\sigma_{i}\left(\nu +\Delta \nu \right),\;\;\;i=1,\ldots ,7\;.$$

In Equation (9) we introduced a shift δν that corresponds to the small permanent shift associated to the damaged state. Then we form the correlation matrix using $\tilde{\Sigma }\left(\nu \right)$ instead of Σ(ν) in Equation (5) and apply the SM to this modified correlation matrix.

**Figure 7:**
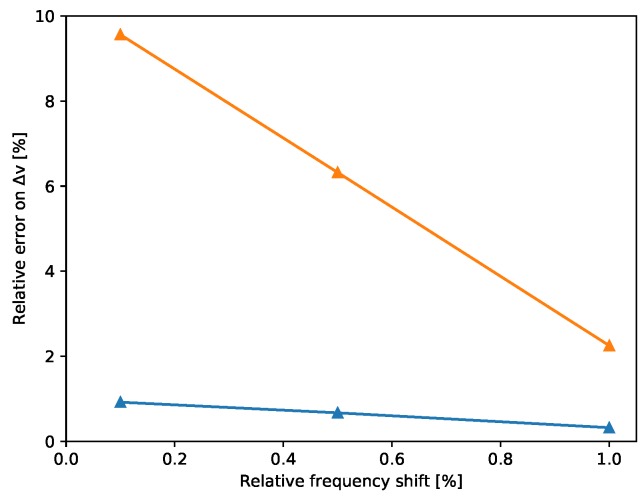
Relative error on Δν when recovering frequency shifts from 0.1% to 1% using either 1 day (orange) or 1 week (blue) as current quantity.

To determine with what accuracy we can detect different levels of frequency shifts in the natural frequencies, we carry out a sensitivity analysis. This consists in manually imposing a frequency shift as described above and then calculate the error of the SM method on recovering the imposed frequency shift. We perform this analysis for frequency shifts from 0.1% to 1% using both 1-day and 1-week measurements to define the current quantity. The results are illustrated in Figure 7, where we observe that the estimation is good for both cases with relative errors below 10%. As expected, the accuracy increases by increasing the time window used for the current quantity. Also, larger frequency shifts can be determined with higher accuracy. Noting that a frequency shift of 1% can be recovered with a relative error less than 4%, we deduce that we can trust our methodology for recovering frequency shifts as small as 1% when using 1-day measurements as current quantity.

**Figure 8:**
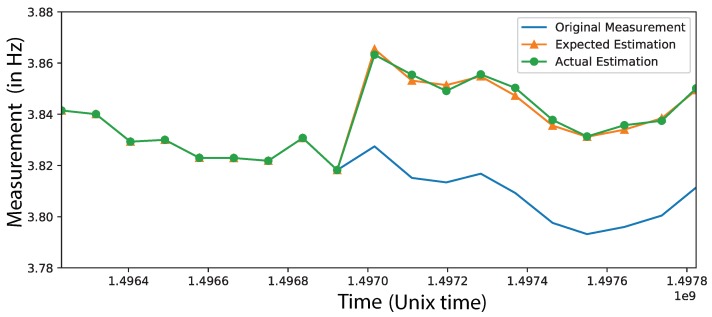
The estimation of first mode using a 24-hour time window for the current quantity and imposing shift of 1%. With orange we plot the exact imposed shift and with green we plot the estimation provided by the SM. We observe very good agreement.

To visualize the performance of SM, we insert a 1% permanent frequency shift at the measurements shown in Figure 6 and try to recover it. The results for the first mode are illustrated in Figure 8. We observe a very good agreement between the estimated and the exact frequency shift indicating that indeed SM can be used in SHM.

Using a simplified numerical model we will provide in the next section possible damage scenarios that correspond to a frequency shift of 1%.

### 3.5. The Estimated Mode Shapes

**Figure 9:**
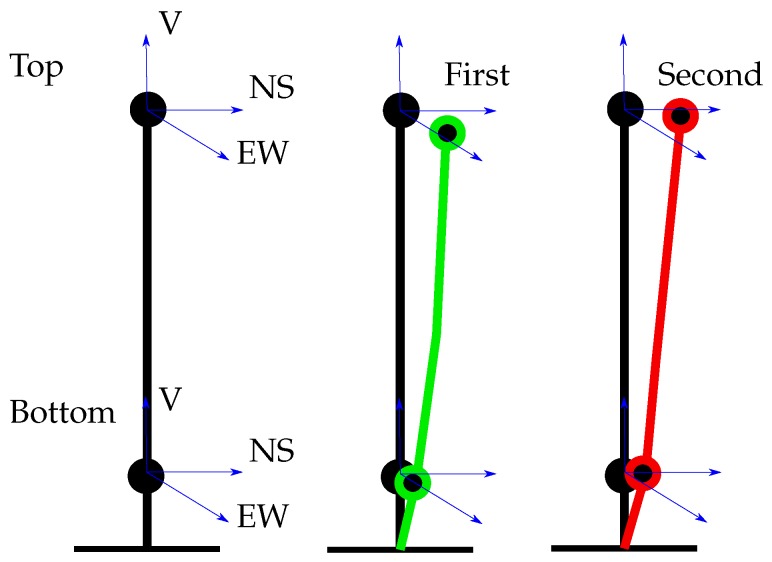
Measured degrees of freedom and schematic illustration of the first two eigenmodes.

Studying the modal values and their variations over time is not always enough for a complete understanding of what the recovered modes actually represent. The whole picture comes from studying the mode’s shape as well. Using the SVD (5) we compute the singular vector corresponding to the largest singular value for the reference quantity at the identified structural frequencies reported on Table 1. The results for the first four modal shapes are reported in Table 2 while the respective degrees of freedom, are shown in Figure 9 together with a schematic illustration of the first two eigenmodes.

## 4. Model Recalibration

In this section we describe the approach we have adopted in order to define a simple yet satisfactory model of the structure under consideration. Finite element simulation of a very similar lighthouse structure in Chania, an almost 80 km neighbor city west of Rethymno, has been accomplished in the past and presented in the literature [31]. The models used there were quite detailed ones with 3D simulations taking place. In the current work we have been experimenting with simpler and more adaptive 1D models with which parametric studies could be easily accomplished, and advanced techniques (minimization, transient analyses) could be more efficiently implemented. The objective here is to construct such simple models having the necessary dynamic properties and still being able to give results in near real time by simulations executed in embedded electronic devises. For that a Java finite element library together with other necessary units (e.g., Apache Maths Common) have been utilized in the development environment of SDE [32]. The simplest model that might be used for such structures is a cantilever beam, dynamic properties of which, in terms of the eigensystem, have been considered in Section 3.3.1. Here we use such a model in combination with the finite element method and appropriate calibration.

### 4.1. Modal Characteristics Consideration

Estimated mode shapes using the acquired data of lighthouse response (see Table 2) indicate the possible coupling of bending to torsional modes. That was expected since cross section asymmetric geometry and/or non-homogeneity of material distribution on the cross section imply coupling of bending and torsional vibrations, since shear and mass center do not coincide over the height. More generally, when geometric, shear and mass centers of the cross sections of a uniform beam do not coincide then coupling of flexural, torsional (including possible warping) and tensional vibration modes occurs [33]. Yet, that coupling might have mostly appeared because of sensors’ positioning. Furthermore, as mentioned before in this work, due to lack of sufficient data, we do not consider any possible torsional and/or tensional motion and we confine ourselves to the study of structure’s bending as a cantilever beam system.

Therefore, we proceed by defining two independent cantilever beam systems, avoiding in such a way the necessity of some 3D model of the beam. The first refers to the East–West and the second to the South-North direction.

#### 4.1.1. Model Calibration as A Minimization Problem

Here we present the model updating procedure. The parameters we search for are the elasticity and inertia distribution over the length of the structure as represented by the elasticity modulus and mass density. We have admitted the quite reasonable assumption of common distribution for both elasticity and inertia.

The methodology we setup is a sensitivity-based element-by-element updating procedure [34]. We use some piecewise linear distribution $\psi_{s}\left(x\right)$ as:

(10) $$\begin{array}{cc} \psi_{s}\left(x\right)= & \left(H\left[x-x_{s-1}\right]-H\left[x-x_{s}\right]\right)\frac{x-x_{s-1}}{\Delta x}+\left(H\left[x-x_{s}\right]-H\left[x-x_{s+1}\right]\right)\frac{\Delta x-x-x_{s}}{\Delta x}, \end{array}$$

where x∈[0,L], $\Delta x=x_{s}-x_{s-1}$ the length of each section and H(x) the Heaviside step function. Then, both elasticity modulus and mass density distributions might be given as:

(11) $$\begin{array}{c} E\left(x\right)=E_{0}\sum_{s=1}^{S}a_{s}\psi_{s}\left(x\right)\;\mathrm{and}\;\rho \left(x\right)=\rho_{0}\sum_{s=1}^{S}a_{s}\psi_{s}\left(x\right). \end{array}$$

Here, we assume ten sections of linear distribution of material properties defined by the coefficients $a_{s}$ which are actually the unknown variables of the minimization problem. Having defined these coefficients $a_{s}$ we also establish the material elasticity and inertia properties distribution.

The objective function *f* to be minimized over the *S* design variables $a_{s}$ might be given as,

(12) $$\begin{array}{c} f\left(a_{s}\right)={\left(\nu_{1}\left(a_{s}\right)-{\tilde{\nu }}_{1}\right)}^{2}+{\left(\frac{\nu_{2}\left(a_{s}\right)}{\nu_{1}\left(a_{s}\right)}-{\tilde{r}}_{21}\right)}^{2} \end{array}$$

accompanied by simple bound constraints on $a_{s}$. We take advantage of the powerful limited-memory BFGS gradient algorithm [35]. The necessary derivative of the objective function *f* with respect to $a_{s}$ is given by,

(13) $$\begin{array}{c} \frac{\partial f}{\partial a_{s}}=2\left(\nu_{1}-{\tilde{\nu }}_{1}\right)\frac{\partial \nu_{1}}{\partial a_{s}}+\frac{2}{\nu_{1}^{3}}\left({\tilde{r}}_{21}\nu_{1}-\nu_{2}\right)\left(\nu_{2}\frac{\partial \nu_{1}}{\partial a_{s}}-\nu_{1}\frac{\partial \nu_{2}}{\partial a_{s}}\right), \end{array}$$

where $\frac{\partial \nu_{i}}{\partial a_{s}}$ denote the sensitivity indexes for the eigenfrequencies. For the dependence of a given eigenfrequency $\omega_{i}$ = $2\pi \nu_{i}$ on design variable $a_{s}$ we get the explicit form [26,36],

(14) $$\begin{array}{c} \frac{\partial \omega_{i}}{\partial a_{s}}=\frac{1}{2\omega_{i}m_{i}^{*}}\phi_{i}^{T}\left(\frac{\partial K}{\partial a_{s}}-\omega_{i}^{2}\frac{\partial M}{\partial a_{s}}\right)\phi_{i} \end{array}$$

where $\phi_{i}$ the *i*-th and $m_{i}^{*}$ the respective generalized mass for that mode given by the product $\phi_{i}^{T}M\phi_{i}$. Sensitivities of stiffness and mass matrices can be explicitly expressed on parameters such as the cross section area *A*, moment of inertia *J*, elasticity modulus *E* and mass density ρ among others. Sensitivity matrices with respect to such parameters are given in the literature (e.g., for the case of Euler-Bernoulli beam [34]). However, as it can be seen in Equation (14), we need to compute the sensitivities $\frac{\partial K}{\partial a_{s}}$ and $\frac{\partial M}{\partial a_{s}}$, of stiffness and mass matrix, respectively. Therefore, considering the chain rule, we get

(15) $$\begin{array}{cc} \frac{\partial \omega_{i}}{\partial \alpha_{s}} & =\frac{\partial \omega_{i}}{\partial E}\frac{\partial E}{\partial \alpha_{s}}+\frac{\partial \omega_{i}}{\partial \rho }\frac{\partial \rho }{\partial \alpha_{s}}\\ & =\frac{1}{2\omega_{i}m_{i}^{*}}\phi_{i}^{T}\left(\frac{\partial K}{\partial E}-\omega_{i}^{2}\frac{\partial M}{\partial E}\right)\phi_{i}\frac{\partial E}{\partial a_{s}}+\frac{1}{2\omega_{i}m_{i}^{*}}\phi_{i}^{T}\left(\frac{\partial K}{\partial \rho }-\omega_{i}^{2}\frac{\partial M}{\partial \rho }\right)\phi_{i}\frac{\partial \rho }{\partial a_{s}} \end{array}$$

with the sensitivities of stiffness and mass matrices over elasticity modulus and mass density parameters computed using the finite element method and $\frac{\partial E}{\partial a_{s}}$, $\frac{\partial \rho }{\partial a_{s}}$ depending only on geometrical features and being easily obtained from Equation (11).

**Figure 10:**
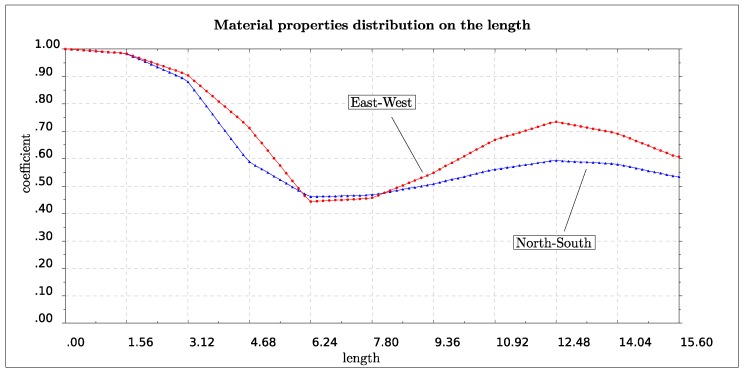
Material properties (elasticity modulus, mass density) nondimensional distribution (vertical axis) with the height (horizontal axis) of the lighthouse.

We consider the two independent cantilever beams for the EW and SN directions respectively defining the finite element model using two hundred elements of equal length, and we set $E_{0}$ = 5.31 GPa and $\rho_{0}$ = 2.5 tn/m${}^{3}$. Using Table 1, the target values for the EW model are ${\tilde{\nu }}_{1}$ = 3.860 Hz and ${\tilde{r}}_{21}$ = 20.859/3.860. For the NS model these values are asked to be ${\tilde{\nu }}_{1}$ = 4.105 Hz and ${\tilde{r}}_{21}$ = 21.190/4.105, respectively. Results for the distribution of elasticity modulus and mass density for both EW and SN models are shown in Figure 10, where we plot the coefficient over the length of the structure with which we should multiply $E_{0}$ and $\rho_{0}$ in order to obtain the target values for each model.

#### 4.1.2. Possible Damage Profiles

**Figure 11:**
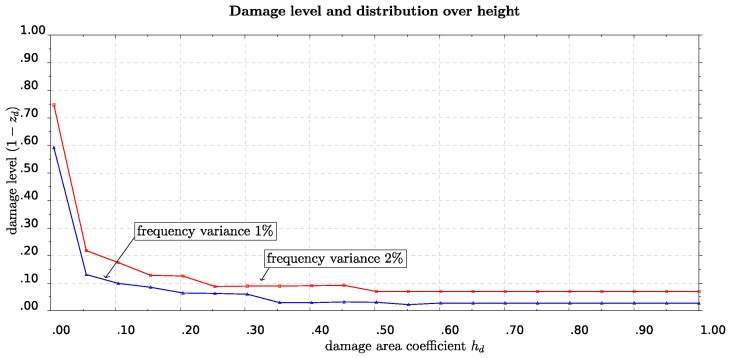
Combination of damage level *z* with damaged area length $h_{d}L$, for variation of eigenfreqency Δν = 1% and 2%.

As it has been shown in Section 3.4, the SM methodology is able to detect one percent variation of the eigenfrequencies. Here we present some possible damage profiles corresponding to that level of variation of eigenfrequencies. In order to do so we assume a variable damage profile of some damage starting from the base of the lighthouse and possibly spreading towards the top. It is worth mentioning that the case of localized severe damage to some restricted area near the base could occur because of an earthquake, while a distributed over the length moderate damage could be the result of some corrosion process (e.g., alveolization, the mechanical action of salt crystallization and action of wind) [37]. To define the damage height we use the coefficient $h_{d}$ taking values from zero up to one and corresponding to zero height, measuring from the base of the lighthouse, up to the total height of it. For this damage area we define a second coefficient $z_{d}$ with which the moment of inertia for cross sections belonging to that damaged area is multiplied. This coefficient $z_{d}$ takes values from one which stands for fully operating cross section up to zero which means totally damaged cross section. The use of this damage variable is in accordance with instructions for moment of inertia reduction because of structural strength deterioration due to cracking. We again solve a minimization problem where, for some specific damage area height $h_{d}$, we ask for the value of the damage variable $z_{d}$ such that the variation of the first or second eigenfrequency is of a specific given level (e.g., 1%–2%). The results are shown in Figure 11, where we present curves of constant frequency variance for one and two percent, respectively. The horizontal axis stands for the length of the damaged area measured from the bottom up to the total height. The vertical axis is the damage level of this area, represented by the coefficient $1-z_{d}$ that takes values from 0 (fully operative) to one (totally damaged). We observe in Figure 11 that in order to have a frequency reduction of 1%, a damage level of 0.6 (corresponding to $z_{d}$ = 0.4) is localized in a small area from the bottom, at about 0.5% of the total length. We also observe that to get a frequency reduction of 2% for the same length of damaged area the respective damage level should be higher, having a value at about 0.75, corresponding to damage variable $z_{d}$ = 0.25.

### 4.2. Time History Consideration

**Figure 12:**
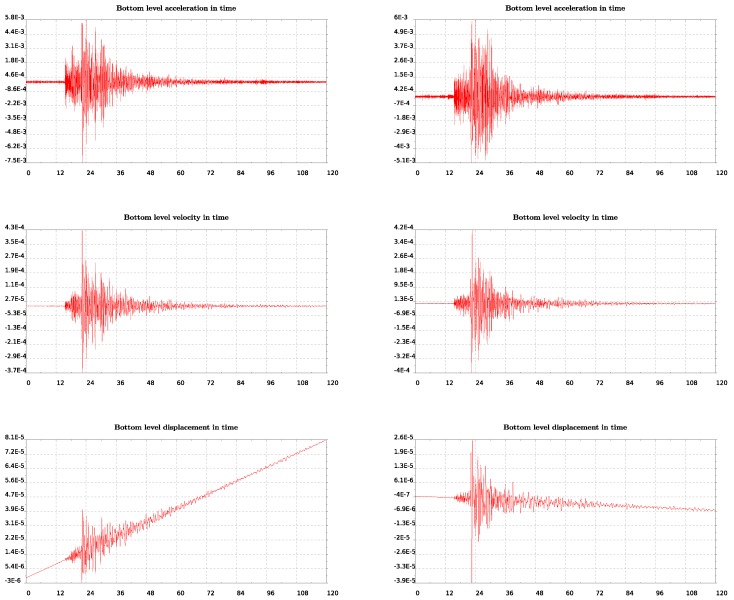
From top to bottom, recorded acceleration integrated in time to give velocities and displacements. On the left column East–West while on the right North–South components are plotted.

In this last section we try to further validate our model in the time domain taking advantage of a small earthquake that occurred and recorded on June 21st, 2017. We plot in Figure 12 the recorded acceleration integrated in time to get velocities and displacements. The East–West components are shown on the left column while on the right are the North–South ones. It is observed in these plots that spurious drifts have appeared in the integrated velocities and displacements time histories. Since they have appeared in both the lower and upper levels of the structure’s measured response, we keep them as they are for further analysis.

**Figure 13:**
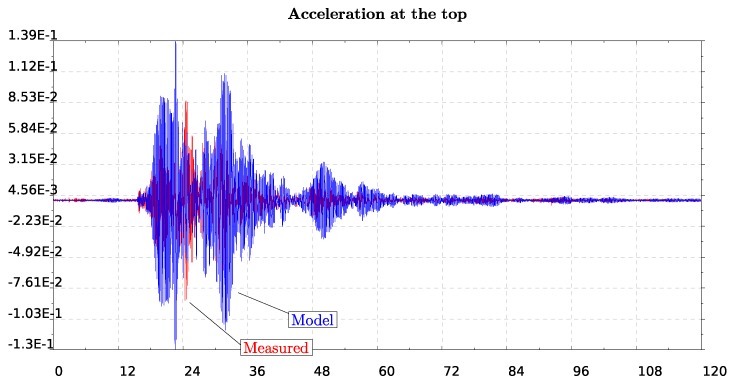

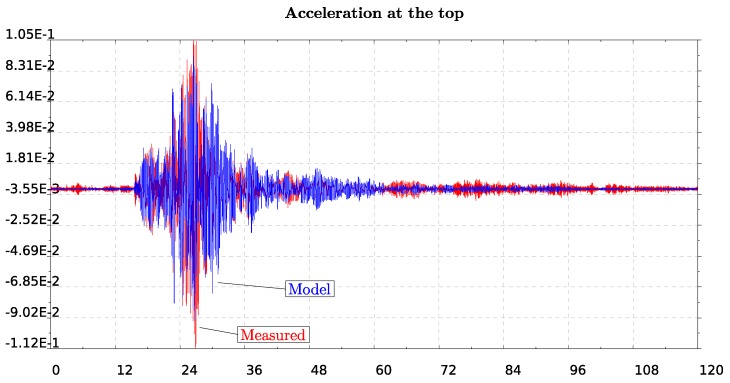
Response of top level acceleration for the East–West model (top plot) and for the North–South (bottom plot), respectively. Both recorded motion (red) and computed by the model (blue) are presented.

In the model, we impose the motion recorded at the lower level of the lighthouse shown in Figure 12 and compute the response at the top level which we further compare with the recorded motion on that level of the lighthouse (see Figure 13). We consider both EW and SN models for which we impose the recorded motion on the lower level. This position is at a height about 10% of the total length while we refer to that as the bottom since it is assumed to be the level on the ground floor of the lighthouse. We time integrate the equation of motion of the finite element system using the β-Newmark method. The critical damping ratio ζ has been estimated to be around 3% using the half power method [29]. We impose motion at the bottom as constraints through the consistent penalty method [38], for which both acceleration and displacement time histories are needed to be given as input.

**Figure 14:**
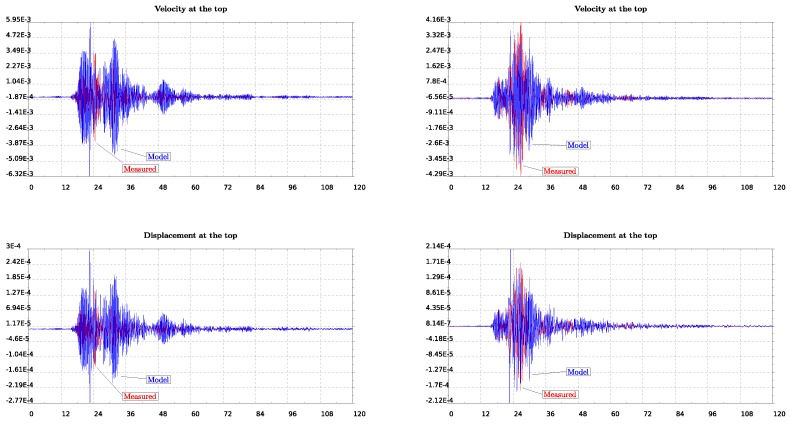
Response of top level velocities (top) and displacements (below) for the East–West model (left) and for the North–South (right), respectively. Both recorded motion (red) and computed by the model (blue) are presented.

In order to compare measured and computed (by the model) results of velocities and displacements at the top of the structure, we proceed by eliminating the drifts. For that, we use a polynomial detrending of the first and second order, for velocities and displacements, respectively. The response at the top of the lighthouse, as computed by the simple (but calibrated) numerical model, is in quite good agreement with the measured response of the structure (see Figure 13 and Figure 14) especially for the North–South component. Note, in particular, that there is an amplification factor of 10 between the bottom and the top acceleration in the measurements, which is well captured by the model.

## 5. Conclusions

The stretching method is a computationally efficient vibration-based technique that uses a very limited number of sensors permanently installed on site to measure operational structural responses for the purpose of damage detection. The preliminary study carried out in this paper on data collected on the Egyptian lighthouse of Rethymnon illustrates the potential of using SM for structural health monitoring of historic monuments. Although our results are quite promising, further effort is needed as regards to both the data acquisition and the model setup. In this study, our model is kept very simple since we do not have so many data to exploit. Note that only two instruments were placed on the lighthouse for a short period of 3 months. A more careful deployment of the instruments (so as to be able to measure axial or torsional modes) on a permanent basis would allow us to carry a more in depth study and illustrate effectively that SM allows for early warning of structural damage.

## Figures and Tables

**Table 1:** The first seven eigenfrequencies estimated from the peaks of $\sigma_{1}^{r}$ (see Figure 3).

Global Index *n*	$\omega_{n}$ (rad/sec)	$\nu_{n}$ (Hz)	Type
1	24.453	3.860	flexural, east-west
2	25.792	4.105	flexural, north-south
3	78.163	12.440	(?)
4	83.290	13.256	(?)
5	86.953	13.839	(?)
6	131.061	20.859	flexural, east-west
7	133.141	21.190	flexural, north-south

**Table 2:** Modal Shapes.

Direction/Position	First Mode	Second Mode	Third Mode	Forth Mode
East–West (EW)/bottom	−0.0801644	−0.0248191	−0.352925	−0.334469
North–South (NS)/bottom	−0.0566074	−0.0120627	−0.314454	−0.29505
Vertical (V)/bottom	−0.00216538	−0.00592483	−0.0370501	−0.121381
East–West (EW)/top	−0.887227	−0.473897	−0.788803	−0.771983
North–South (NS)/top	−0.450108	−0.87642	−0.364553	−0.385618
Vertical (V)/top	−0.024408	−0.0807035	−0.141706	−0.204166
